# Racial health disparities in ovarian cancer: not just black and white

**DOI:** 10.1186/s13048-017-0355-y

**Published:** 2017-09-21

**Authors:** Sanjeev K. Srivastava, Aamir Ahmad, Orlandric Miree, Girijesh Kumar Patel, Seema Singh, Rodney P. Rocconi, Ajay P. Singh

**Affiliations:** 1 Department of Oncologic Sciences, Mitchell Cancer Institute, University of South Alabama, 1660 Springhill Avenue, Mobile, AL 36604-1405 USA; 2 Division of Cell Biology and Genetics, Tatva Biosciences, Coastal Innovation Hub, 600 Clinic Drive, Mobile, AL 36688 USA; 3 Department of Biochemistry and Molecular Biology, College of Medicine, University of South Alabama, Mobile, AL 36688 USA; 4 Division of Gynecologic Oncology, Mitchell Cancer Institute, University of South Alabama, Mobile, AL 36604 USA

**Keywords:** Ovarian cancer, Racial health disparity, Socioeconomic, Epigenetic

## Abstract

Ovarian cancer (OC) is the most lethal gynecological malignancy, which disproportionately affects African American (AA) women. Lack of awareness and socioeconomic factors are considered important players in OC racial health disparity, while at the same time, some recent studies have brought focus on the genetic basis of disparity as well. Differential polymorphisms, mutations and expressions of genes have been reported in OC patients of diverse racial and ethnic backgrounds. Combined, it appears that neither genetic nor the socioeconomic factors alone might explain the observed racially disparate health outcomes among OC patients. Rather, a more logical explanation would be the one that takes into consideration the combination and/or the interplay of these factors, perhaps even including some environmental ones. Hence, in this article, we attempt to review the available information on OC racial health disparity, and provide an overview of socioeconomic, environmental and genetic factors, as well as the epigenetic changes that can act as a liaison between the three. A better understanding of these underlying causes will help further research on effective cancer management among diverse patient population and ultimately narrow health disparity gaps.

## Background

Ovarian cancer (OC) is the most lethal of all gynecologic malignancies [1]. Globally, OC is the 7th most commonly diagnosed and the 8th leading cause of cancer-related mortality among women [2]. According to estimates by the American Cancer Society, 22,440 new OC cases and 14,080 associated deaths will occur in 2017 [1]. It is a deadly disease with no effective screening [3]. Approximately 70% of patients with OC are diagnosed at an advanced stage, with associated poor prognosis, even after aggressive and immediate treatments [4]. Studies over past several years have revealed that besides being highly lethal, OC also disproportionately affects some distinct racial populations, particularly the black women of African American (AA) heritage, as compared to the Caucasian Americans (CA) or white women of European heritage. In fact, even with higher incidence of OC in CA women in the United States, the associated mortality is disproportionately higher in AA women [5], and such disparities are common worldwide [6]. Though the exact causes of racial disparities in OC still remain unclear, they are likely to be multifaceted and may include socio-cultural factors, acquired co-morbid conditions, increased frequency of modifiable risk factors, access to health care, diet and preventive health factors. Emerging data suggests that several biological factors, such as genetic, epigenetic etc., could be more crucial than thought for health disparities in OC incidence and outcome [5, 7, 8]. In this review article, we have discussed, in-detail, various biological and non-biological factors in racially disparate clinical outcomes of OC.

## Disparity in ovarian cancer incidence and mortality

While some progress has been made in OC treatment, it has been observed that AA women with OC are not reaping the same benefits of the advances as CA women. Progress in management of OC patients has improved over time but this progress has been relatively slow for AAs [9]. The all-cause mortality of AA OC patients is 1.3 times higher, as compared to CA OC patients, even when access to care is equal [5]. According to data from SEER database, between the years 1992 and 2008, the five-year survival rate for CA women rose from 40.7% to 45.0%, while the five-year survival for AA women fell from 47.9% to 40.3%. For the years 2006 through 2012, the reported 5 year survival of AA OC patients is relatively poor, compared to CA OC patients, irrespective of cancer stage at the time of diagnosis [1] (Table 1). Although the overwhelming majority of all OC patients are diagnosed at an advanced stage, it has been suggested that AA women bear a greater burden in the late diagnosis than CA women. Increased instances of late diagnosis may be attributed to socio-economic factors that will be discussed later in this article.

**Table 1:** Five-year relative survival rates of ovarian cancer patients in United States, by stage at the time of diagnosis

Diagnosis stage	African American	Caucasian American	All races
Localized	86	93	92
Regional	58	73	73
Distant	21	29	28
All stages	36	46	45

The data is for years 2006 through 2012 [1]

This disparity in survival may also be linked to observed prevalence of comorbidities. Evidence shows that AA women diagnosed with OC are much likely to have hypertension (75.5%), renal disease (58.5%) and cardiovascular disease (63.21%) [10]. Often, AA women, post-treatment, tend to have elevated CA-125 levels [10]. CA-125 is a diagnostic marker for OC and its elevated levels indicate a lingering OC, which could implicate higher recurrence and, possibly, reduced survival. Further, earlier normalization of CA-125 levels during primary chemotherapy for epithelial OC predicts improves progression free survival (PFS), overall survival (OS) as well as platinum sensitivity [11]. For each one-cycle improvement in CA-125 normalization, there is an estimated 3.8 months increase in PFS, 8.6 months increase in OS and 8% increase in platinum sensitivity.

With the realization of race-based OC health disparities [12], efforts are now being made to better understand the etiology and progression of OC in AA populations. For example, a recent study looked at the lifetime number of ovulatory cycles and epithelial OC risk in just AA patients [13]. The study found a positive association between lifetime ovulatory cycles and epithelial OC in AA women, similar to what was already known in CA patients. The association of antioxidant selenium uptake and risk of OC in AA women was also studied in a cohort of 406 AA OC patients and 632 AA healthy (control) women that were age- and site-matched [14]. Consistent with the beneficial effects of antioxidants, women with high selenium intakes were found to be at ~30% lowered risk of OC. Further, oral contraceptive use, pregnancy and breastfeeding were observed to be inversely correlated with OC in AA women, similar to what was already known in CA women [15]. Also, high intake of dietary sugars and glycemic load were confirmed to be associated with higher OC risk in AA women, similar to other populations [16].

## Socioeconomic and other factors contributing to racial health disparities in ovarian cancer

Socioeconomic status (SES) remains a major factor driving OC health disparities. In a study that evaluated 2432 epithelial OC patients (1989 CA vs. 443 AA) in Cook County, Illinois between the years 1998 and 2007, neighborhood SES, particularly the affluence and disadvantage, had profound effect on OC survival [17]. Even after adjusting for factors such as age, tumor characteristics, treatment and year of diagnosis, AAs were much more likely to succumb to OC, compared to CAs. This disparity could be attenuated only if SES factors were factored-in as well. On very similar lines, in a study that evaluated patients from the same locality, but from an early time-period (1994 through 1998), neighborhood disadvantage was still observed as the primary reason for OC-specific survival [18]. In an earlier report [19], AA OC patients were reported to be less likely to undergo surgery and chemotherapy. Unfortunately, the OC health disparity has increased over time and one reason for this increase is the disparate access to treatment, particularly surgery [20]. In a study that evaluated 393 OC patients (68 AA vs. 325 CA), a clear survival disadvantage was observed in AA patients [21]. The AA patients had lower SES, in terms of education, income and property, to start with. However, the survival disadvantage was evident even when controlled for these factors. Progression free survival was only 16 months in AA OC patients, as compared to 27 months in CA OC patients, whereas overall survival was less than half in AA patients (42 months), as compared to CA patients (88 months).

Racial disparity has been noted in the diagnosis of OC [22] with AA women being frequently diagnosed with advanced stage OC. However, interestingly, in one study, no race-based differences in clinical outcome were reported in patients with advanced stage epithelial OC, provided they received similar treatment [23]. While elsewhere, it has also been reported that AA OC patients are much less likely to receive standard guideline-recommended care, as compared to CA patients [24]. Although these studies seem to support the notion that equal access to healthcare is an important factor in determining disparate OC outcomes, it is also possible that this was because of the fact that these studies focused on advanced stage OCs, where the disease was too progressed and aggressive in the patient populations to reveal a meaningful correlation of progression and/or survival with the race. Access to healthcare has often been discussed as an important factor that influences cancer health disparity. In OC, access to health care and its impact on survival of OC patients was studied in a cohort study in Northern California [10]. Since the cohort comprised of members of Kaiser Permanente, it was assumed that the access to health care was uniform for all subjects. Even with equivalent access to health care, AA women had worst survival, which was, in part, due to treatment delay and early discontinuation.

**Fig. 1:**
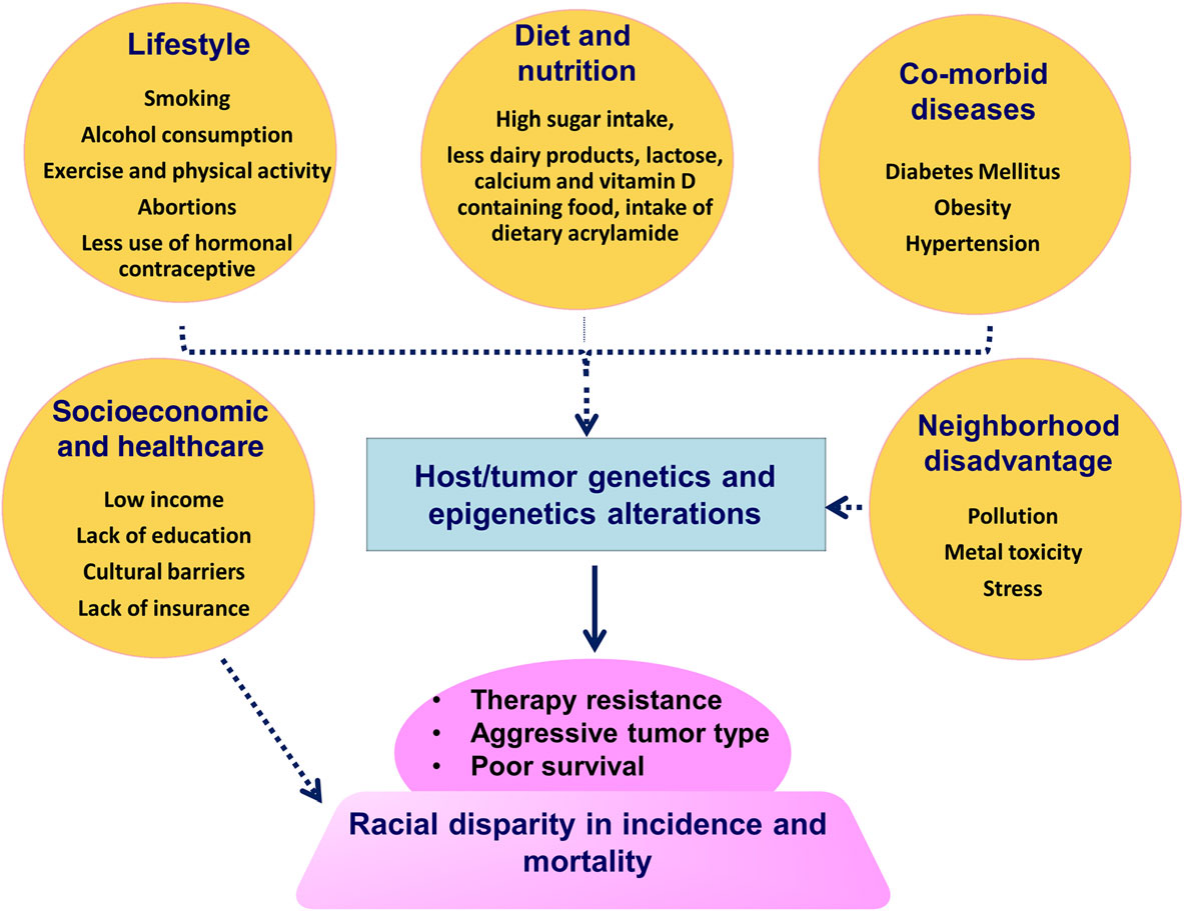
Interplay between various non-biological and biological factors contributing to ovarian cancer racial health disparities. Various non-biological factors such as socioeconomic, lifestyle, dietary habits, neighborhood disadvantage, co-morbid diseases may impact genetic/epigenetic aberrations in both host and tumor resulting in the development of aggressive and therapy resistant phenotypes These factors may be responsible for racially disparate clinical outcomes

Geographical proximity to a tertiary hospital system and travel distance are also factors that play a role in disparate outcome of OC in AA women [25]. Availability of reliable mode of transportation is part of the SES, and it is believed that AA OC patients are much less likely to travel more than 20 miles to a big hospital for treatment, compared to CA patients. While ~22% CA patients would travel more than 20 miles for treatment, only about 14% AA patients would likely travel this distance. This results in increased risk of non-adherent care and the resulting sub-optimal treatment and follow-up. AA patients are also significantly less likely to undergo initial surgical intervention for OC at big, high-volume hospitals, compared to CA patients [26]. Participation of minorities in clinical trials is yet another challenge. It has been reported that the overall enrollment of AA populations in gynecologic oncologic group (GOG) trials is dismal, and is probably going down further, according to the most recent data [8]. In this data, generated from 445 GOG publications between 1985 and 2013, the overall participation of AA patients was just 8%, compared to 83% CA patients. The more disturbing observation was that the situation is probably getting worse as the enrolment of AA patients has gone down 2.8 folds for the years 2009–2013, compared to the enrolment in years 1994 through 2002. The various non-biological factors that contribute to OC health disparities, as discussed in this section above, are summarized in Fig. 1.

## Biological basis of ovarian cancer health disparities

While non-biological factors play an important role in OC health disparity, there is evidence to suggest a critical role of biological factors in OC health disparity as well [6]. In fact, enhanced incidence and mortality in AA women with OC has been observed, as compared to EA counterparts, even when socioeconomic factors have been accounted for. For example, in a study that evaluated 393 OC patients (68 AA vs. 325 CA), a clear survival disadvantage was observed in AA patients [21]. The AA patients had lower SES, in terms of education, income and property, to start with. However, the survival disadvantage was evident even when controlled for these factors. Progression free survival was only 16 months in AA OC patients, as compared to 27 months in CA OC patients, whereas overall survival was less than half in AA patients (42 months), as compared to CA patients (88 months). Possible involvement of biological factors in OC health disparity was also suggested in a study that factored for access to healthcare, and still observed poor survival of AA OC patients, compared to CA patients [10].

Higher adiposity is a known OC risk [27]. Since AA women have disproportionately high rate of obesity [28], a study was carried out to evaluate whether body mass index (BMI) or the weight gain since the age of 18 years can be independent OC risk factors [27]. It was observed that a BMI ≥ 40 posed a significant OC risk, compared to BMI < 25, thus connecting obesity with increased OC risk. Similarly, gain of weight since age 18 also correlated positively with OC risk [27]. In a similar study, where BMI ≥ 30 was considered obese, AA women with higher BMI reported having OC symptoms much before the formal diagnosis [29]. Severe obesity is a critical risk factor for OC [30], underlining the importance of healthy and active lifestyle.

**Fig. 2:**
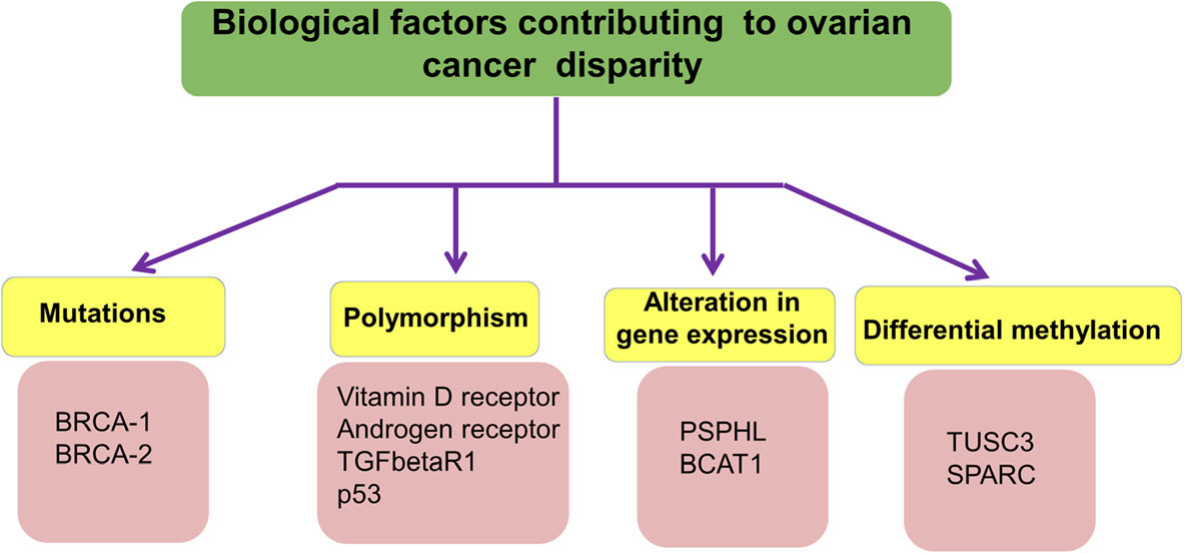
Biological factors associated with ovarian cancer racial health disparities. Mutations, genetic polymorphisms, epigenetic alterations and aberrant gene expressions play important role in ovarian cancer health disparities

Several molecular alterations such as gene-mutations, genetic polymorphism, epigenetic alterations etc. have been associated with OC racial health disparities (Fig. 2). In this section we discuss the role of these molecular factors in the racially disparate outcome of OC.

### Genetic factors associated with ovarian cancer health disparities

The underlying causes for OC health disparities are complex; they are clearly a mixture of biological as well as non-biological factors [31]. Phosphoserine phosphatase like (PSPHL) expression is elevated in AAs, compared to CAs [32]. PSPHL was primarily identified as a differentially over-expressed gene in AA endometrial cancers, relative to CA endometrial cancers. Since both ovarian and endometrial cancers belong to the family of gynecological cancers, the study was extended to evaluate PSPHL expression in AA vs. CA OCs as well. Similar to endometrial cancers, PSPHL was found to be elevated in AA OCs, as compared to CA OCs. Interestingly, PSPHL levels are relatively higher in AA breast tumors than the CA breast tumors [33]. Health disparities in breast cancer are well documented [34, 35] and the study on PSPHL in breast tumors [33] identified partial deletion (30Kb long fragment) of chromosome 7p11 in CA women, which led to attenuation of expression of PSPHL in CA breast tumors. No such mechanism was proposed for observed disparate expression of PSPHL in OCs of AA vs. CA origin, and would be interesting to elucidate.

AA women are at relatively higher risk of OC because of higher rates of BRCA1 and BRCA2 mutations, as compared to other racial/ethnic populations [36, 37]. This represents an interesting evolution in our understanding of BRCA mutations in AA women because early studies on the subject seemed to suggest the opposite; that AA women harbor significantly less BRCA mutations, compared to CA women [3]. BRCA1 and BRCA2 are tumor suppressors that play a role in repair of damaged DNA. Inherited mutations in BRCA1 and BRCA2 often result in breast and/or OC. It has been estimated that the patients with BRCA1 mutations have 39% while those with BRCA2 mutations have 11% risk of developing OC by an age of 70 years [38].

The connection between inflammation and cancer is well known [39], and, recently, it has been suggested that pro-inflammatory diet leads to increased risk of epithelial OC in AA women [40]. The study calculated ‘dietary inflammatory index’ through a questionnaire that focused on dietary intake in the year prior to diagnosis of OC [40]. Women that consumed the most pro-inflammatory diet were at significantly higher risk of epithelial OC. Inflammation is known to play a role in OC onset and progression [41]. The pro-inflammatory cytokines produced within the tumor microenvironment help ovarian tumors proliferate as well as evade chemotherapy [42]. Studies document that high intake of carbohydrate-rich foods may result in the production of IGF-1 that is known to promote ovarian tumorigenesis via stimulation of hormones such as androgens. Moreover, alterations in glucose levels could induce oxidative DNA damage [16]. Analgesic medications also seem to play a role in OC risk. AA women taking aspirin for prevention of cardiovascular diseases or the non-aspirin nonsteroidal anti-inflammatory drugs for arthritis had 44% and 26% lower risk of epithelial OC, respectively [43].

Vitamin D is another factor that is increasingly being correlated with cancer health disparities [44]. The darker skin pigmentation in AA populations results in significantly lower serum vitamin D levels, and since vitamin D levels inversely correlate with multiple cancers [44], lower serum vitamin D levels are observed in patients diagnosed with different cancers, including OC. It has been suggested that daily intake of vitamin D, along with calcium, can reduce the cancer risk in women by 60% [45]. Further, for every 10 ng/mL increase in the levels of vitamin D, the cancer risk decreases by 35% [45]. As a direct connection between OC and vitamin D, it has been reported that OC patients are four times more likely to have lower serum vitamin D levels, as compared to healthy controls [46]. Studies have shown that vitamin D inhibits cancer cell proliferation by inducing cell cycle arrest at G1 phase via up-regulation of expression of CDKIs p21WAF 1/ Cip 1 and p27Kip [47]. A vitamin D response element is also known to be present in the promoter region of p21 gene, suggesting that vitamin D may directly cause the transcriptional activation of p21 [48].

Gene polymorphisms have also been implicated in disparate OC in AA women. Vitamin D receptor polymorphism, particularly at a minor allele rs7975232, has been linked to higher risk of epithelial OC in AA women [49]. SNP rs7305032, in close proximity to this allele, correlated with nearly two-folds increased risk of invasive epithelial OC in AA women [49]. Similarly, CAG repeat length polymorphism in exon 1 of androgen receptor gene was observed to increase risk of OC in AA, with, interestingly, no such correlation in CA [50]. In one of the earliest studies on the topic, racially disparate polymorphism of tumor suppressor gene p53 was suggested [51]. Codon 72 of p53 gene has arginine and proline allelotypes. The arginine allelotype is more frequent in CAs while the proline allelotype is more frequent in AAs [51]. In a study that evaluated the 6A allele of type I transforming growth factor beta receptor 1 (TGFβR1), no increased OC risk was associated with the allele [52]. However, significant racial differences in the frequency of this allele were observed, with the allele being relatively infrequent in AA women (2.4%), compared to CA women (10.7%). A more thorough study would be needed to better understand the link between this TGFβR1 allele and racial disparity in OC.

### Epigenetic changes in ovarian cancer health disparities

Epigenetic changes are known to function as liaisons between socioeconomic factors and the genome; they keep accumulating and affecting the epigenome in various racial/ethnic groups, resulting in disparate and poor cancer outcomes [35]. A functional role of epigenetic changes in aggressive high-grade serous OC has been described, leading to resistance against platinum-containing chemotherapy [53]. AA OCs are much more aggressive, and associated with poor overall survival, compared to CA OCs. This suggests the possibility of epigenetic modifications as factors that play a role in OC health disparity, an idea that has not yet been tested.

As mentioned above, endometrial cancers and OCs belong to the same family of gynecological cancers. Incidentally, there is evidence in the literature suggesting an epigenetic basis of health disparity in endometrial cancers [35], particularly, differential ribosomal DNA methylation in AA vs. CA patients [54]. This raises the hope that the differential methylation, as observed in drug-resistant OCs [53] might also be relevant in OC health disparity. This speculation is further fueled by some indirect evidences that link epigenetically-regulated genes in OC with health disparities in other cancers. For example, methylation of *TUSC3* (tumor suppressor candidate 3) [55] has been linked to poor disease-free survival of OC patients. *TUSC3* was part of a gene signature evaluated for differential methylation and expression in AA vs. non-CA breast tumors [56]. Further, silencing of *SPARC*, through hypermethylation, has been reported in OC [57]. This gene was part of a signature that was investigated for putative role in prostate cancer health disparity. SPARC has been reported to be frequently down-regulated in cancer cells [58]. It is silenced through promoter methylation in metastatic and aggressive cancer cells [59]. Finally, *BCAT1* (branched chain amino-acid transaminase 1) is reportedly over-expressed in ovarian tumors [60], possibly through a mechanism involving hypomethylation [61]. *BCAT1* was one of the top up-regulated genes in AA colorectal cancer patients-derived samples, as compared to samples from CA colorectal cancer patients. In light of these observations, it might be worthwhile to evaluate these genes, as a starting point, for their potential disparate expression and a role in OC health disparity.

Regulation of gene expression by differential methylation is a classic example of epigenetic event. However, regulation of genes through miRNAs is also within the broader definition of epigenetic changes. A number of miRNAs have been reported deregulated in OC models with potential role as diagnostic and/or prognostic markers [62]. Also, miR-152 has been shown to regulate DNMT1 (DNA methyltransferase-1) [63], an enzyme that actively influences the overall methylation status by transferring methyl groups to its targets. miR-152 is also linked to cancer health disparity as it was significantly down-regulated in about ~50% of AA prostate cancer tissues, compared to 35% of CA samples [64].

### Interplay of genetic and environmental factors in ovarian cancer and associated health disparities

Environmental factors influence genetic factors, and such interactions lead to onset of human cancers. Risk of OC has been linked to several environmental factors [65, 66]. Although occupational exposures and environmental factors seem to correlate with increased OC risk, epidemiological analyses have often failed to establish a positive link [67, 68]. This has been blamed on cohort size, statistical power and several other factors.

One environmental factor that has been linked to increased OC risk is the use of pesticides and herbicides [66]. This is particularly concerning in developing countries where stable organochlorine pesticides represent a bulk of pesticides in use [69]. These pesticides persist in environmental because they are not easily biodegradable and the half-lives run in decades. Their carcinogenic activity is related to estrogen-mimicking property [69]. While indiscriminate use of such harmful compounds is relatively more prevalent in under-developed and developing countries, even the developed countries are not immune to this environmental risk of OC. For example, in the US, pesticide atrazine has been detected in public water supplies in Iowa [70]. It is documented that atrazine causes phosphorylation of ERK and induces expression of oncogenes such as cyclin A, progesterone receptors, c-fos etc. to promote the proliferation of cancer cells [71]. Levels of nitrates in the drinking water were linked to an increase in OC risk, especially among the post-menopausal women, in the Iowa Health Women’s study [72]. CYP2E1 gene mutation is reported to be associated with cytotoxicity and DNA damage as a result of nitrosamines [73]. Based on this, it could be speculated that there may be an association between high nitrate containing drinking water and genetic variation in CYP2E1, which may present increased risk of OC in AA women.

Talc, consisting of magnesium silicate, is another environmental factor with possible carcinogenic implications. The association between talc (talcum) and OC has been controversial, and a subject of legal battles [74]. When applied to genital areas, diaphragms or sanitary napkins, it can possibly cause OC. A pooled analysis of 8525 patients observed a moderate risk of OC in patients with reported genital powder use [75], with positive correlation between powder use and elevated risk of individual OC subtypes. A prospective study that followed 61,576 post-menopausal women for 12.4 years, however, could not link talc use with OC risk [76]. The results were confirmed by another independent study which found douching, and not talc use, as a risk factor for OC [77]. A recent meta-analysis, however, confirmed risk of OC from genital talc use with a statistically significant risk of serous OC [78]. It has been suggested that the risk of OC from genital talc use is rather complex, and involves many considerations, such as menopause status, hormone therapy, weight, smoking etc. [79]. Harmful effects of talc include elevated immunoglobulins and heat shock proteins [80]. In a gene-talc interactions study performed in OC, it was observed that women exhibiting specific genetic polymorphism in *GSTM1* and *GSTT1* may have a higher risk of serous invasive OC, associated with genital talc use [81]. Functional role of *GSTM1* and *GSTT1* in the metabolism of carcinogens and reactive oxygen species has been reported [82]. Thus, although, environmental factors have been suggested to influence OC, and even confirmed by a few analyses, the topic remains controversial with individual analyses marked by limitations that prevent an objective assessment and a definite conclusion. While designing future studies, it would also be interesting to evaluate the role of several environmental risk factors in OC cancer health disparities.

## Conclusions and perspectives

The existence of OC health disparities is undeniable, as per the evidence that has accumulated over last decade or so. For records, it needs to be acknowledged that it’s not just the AA women, but OC health disparities are evident in other populations as well, such as, Hispanics and Asian/Pacific Islanders [10, 26, 83]. What is not clearly understood is the basis of such disparities. Non-biological factors, particularly the socioeconomic ones, make sense but emerging literature clearly hints at existence of defining biological factors as well. While non-biological factors might appear to be simple and straight-forward, a closer look reveals that OC health disparities involving SES factors such as neighborhood-related, income, access and affordability of treatment etc. are not easy to be addressed. A number of steps need to be taken to tackle these issues [84]. These will invariably involve some grass-root level efforts with involvement of community outreach programs, counseling initiatives and, possibly, social media. The establishment of partnerships such as Georgia Breast Cancer Genomics Health Consortium to assist young AA women at increased risk of hereditary breast and OC is a step in this direction [85]. In addition to the various non-biological and biological bases of OC health disparities, there is an added burden of factors that are difficult to classify one way or the another. An example is the suggestion that more prevalent use of genital powder in AA women is a risk factor for epithelial OC [86]. Thus, an interplay of biological as well as non-biological factors, with some role of environmental factors, is possibly a more acceptable explanation for observed OC health disparities. This brings in epigenetic factors that provide a logical connection between biological, non-biological as well as environmental factors. When compared to other cancers with documented health disparity, the literature on OC is still in its infancy. However, we need to remind ourselves that OC is the most lethal gynecological malignancy. It’s about time that more sincere efforts are made to better understand and target OC health disparities, so that the dismal screening and outcome can be substantially improved.

## Abbreviations

AA: African American
BCAT1: Branched chain amino-acid transaminase 1
BMI: Body mass index
CA: Caucasian American
CDKIs: Cyclin dependent kinase inhibitors
CYP2E1: Cytochrome P450 family 2 subfamily E member 1
DNMT1: DNA methyltransferase-1
GSTM1: Glutathione s-transferase Mu 1
GSTT1: Glutathione s-transferase theta-1
miRNA: MicroRNA
OC: Ovarian cancer
OS: Overall survival
PFS: Progression free survival
PSPHL: Phosphoserine phosphatase like
SES: Socioeconomic status
TGFβR1: Transforming growth factor beta receptor 1
TUSC3: Tumor suppressor candidate 3
